# Tamarind-Derived Trypsin Inhibitor as a Potential Therapeutic Agent for Obesity: Evidence from In Vitro and Zebrafish Models

**DOI:** 10.1021/acsomega.5c05693

**Published:** 2025-08-15

**Authors:** Aline Lopes Marques de Sousa, Raphael Paschoal Serquiz, Ana Carolina Luchiari, Tayana Cabral Figueiredo, Aslan Costa Trajano, Emilly Guedes Oliveira e Silva, Alexandre Coelho Serquiz, Ana Emília Nascimento Lemos, Elizeu Antunes dos Santos, Juliana Kelly da Silva Maia, Ana Heloneida de Araújo Morais

**Affiliations:** † Federal University of Rio Grande do Norte, Center for Healthy Sciences, Postgraduate Nutrition Program, Natal, Rio Grande do Norte 59078-970, Brazil; ‡ Federal University of Rio Grande do Norte, Biosciences Center, Department of Biochemistry, Natal, Rio Grande do Norte 59078-970, Brazil; § Federal University of Rio Grande do Norte, Biosciences Center, Department of Physiology and Behavior, Natal, Rio Grande do Norte 59078-970, Brazil; ∥ Federal University of Rio Grande do Norte, Center for Healthy Sciences, Course of Nutrition, Natal, Rio Grande do Norte 59078-970, Brazil; ⊥ Federal University of Paraíba, Center for Healthy Sciences, Department of Nutrition, João Pessoa, Paraíba 58051-900, Brazil; # Federal University of Rio Grande do Norte, Biosciences Center, Postgraduate Program in Biochemistry and Molecular Biology, Natal, Rio Grande do Norte 59078-970, Brazil; ¶ Federal University of Rio Grande do Norte, Biosciences Center, Postgraduate Program of Psychobiology, Natal, Rio Grande do Norte 59078-970, Brazil

## Abstract

Obesity is a multifactorial disease, with numerous therapeutic targets. In this context, various peptides and proteins have been the focus of research due to their ability to influence body weight regulation. This study aimed to investigate the effect of trypsin inhibitor isolated from tamarind seeds (TTI) on lipase activity, both in vitro and in vivo, using the zebrafish (*Danio rerio*) as an animal model. TTI was first isolated through affinity chromatography using trypsin-Sepharose 4B, followed by characterization of its antitryptic activity, protein quantification, and molecular mass. An in vitro inhibition assay was then performed against porcine pancreatic lipase to determine the half-maximal inhibitory concentration (IC_50_) and inhibition constant (*K*_i_) of TTI. TTI inhibited the lipase activity by 83%. The IC_50_ was estimated to be 1.59 × 10^–9^ mol L^–1^, and the *K*_i_ was 2.38 × 10^–8^ mol L^–1^, indicating that TTI acts as a reversible noncompetitive inhibitor. The preclinical study involved diet-induced obese zebrafish. The fish were divided into five groups: eutrophic and normofed animals without treatment; obese and hyperfed animals without treatment; obese and hyperfed animals treated with Orlistat (50 mg/kg Orlistat); obese and hyperfed animals treated with TTI (25 mg/L TTI); and obese and normofed animals treated with TTI (25 mg/L TTI). After 10 days of treatment, the groups were evaluated for lipase activity, body weight, and lipid profile. Results showed that neither Orlistat nor TTI inhibited lipase activity under the tested in vivo conditions. However, TTI-treated hyperfed and normofed animals showed a significant reduction in body weight compared with the control groups (obese and hyperfed animals without treatment and obese and hyperfed animals treated with Orlistat). Moreover, HDL concentrations were significantly higher in the TTI-treated groups compared with all other groups. Thus, TTI represents a promising strategy for the treatment of obesity and the prevention of dyslipidemia, opening new avenues for exploring its potential benefits against other obesity-associated comorbidities.

## Graphical abstract

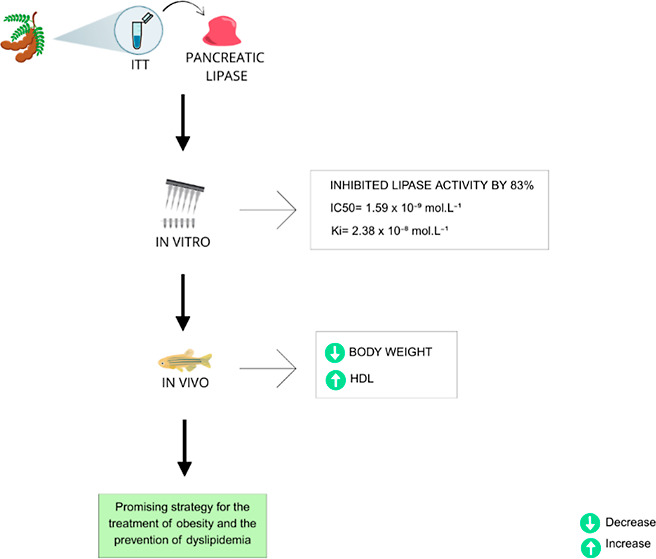

## 1 Introduction

Obesity is a global, multifactorial disease characterized by abnormal or excessive fat accumulation, which can harm health and increase the risk of various conditions, including type II diabetes mellitus, cardiovascular diseases, metabolic syndrome, and cancer.^1^ Recent research has also highlighted the spleen’s role in the pathophysiology of obesity, particularly through the production of pro-inflammatory cytokines and chemokines, which contribute to comorbidities such as nonalcoholic fatty liver disease (NAFLD) via the so-called liver–spleen axis.^2^ Furthermore, obesity has been associated with a higher risk of developing severe cases of COVID-19.^1,3^

Traditionally, obesity has been attributed to an imbalance between caloric intake and energy expenditure.^4^ However, it is now understood that the development of obesity results from a complex interaction of biological, environmental, and psychosocial factors.^1^

Since 1980, the prevalence of excessive weight gain has doubled worldwide, and currently, about one-third of the global population is classified as obese or overweight.^5^ This increase has been observed in both men and women, showing a continuous upward trend.^1^ According to the World Obesity Federation, it is estimated that by 2035, 41% of the adult population in Brazil will be classified as obese.^6^ Due to its growing prevalence, significant health impacts, and associated medical costs, obesity has become a major public health issue.^7^

The Body Mass Index (BMI) above 30.0 kg/m^2^ ^5^ is the primary diagnostic tool for obesity, according to the guidelines of the World Health Organization (WHO). However, this criterion has significant limitations, as it solely reflects overall adiposity and does not distinguish between fat distribution or body composition.^8^ Therefore, additional parameters should be considered to more accurately define overweight and obesity.^9,10^ These include waist circumference, waist-to-hip ratio, and waist-to-height ratio. When possible, the use of direct methods to assess fat mass, such as DEXA or bioimpedance, is recommended.^11^

In the management of obesity, current guidelines define clinically significant weight loss as a reduction of at least 5% of initial body weight, which is associated with improvements in cardiometabolic risk factors.^12^ However, a major challenge is that individuals with obesity often have poor long-term adherence to lifestyle changes, leading to limited weight loss through nonpharmacological interventions.^13^ Consequently, pharmacological treatment is recommended for patients who do not achieve adequate weight loss through lifestyle modifications alone.^12,14^ Nevertheless, the undesirable side effects associated with the use of antiobesity drugs sparked a growing interest in natural alternatives containing bioactive compounds that can improve disease outcomes while presenting fewer adverse effects.^15,16^ Among these approaches, the effects of peptides with lipase-inhibiting properties have been increasingly studied.^17–19^

Lipases are water-soluble enzymes that are responsible for the hydrolysis of dietary fats. They convert triglycerides into monoacylglycerols and free fatty acids, facilitating the absorption of these macronutrients by enterocytes.^20^ Inhibiting lipase is considered a therapeutic target for obesity, as its inhibition slows the absorption of fatty acids into systemic circulation and adipocytes, directly addressing the root cause of obesity.^21,22^

A significant source of bioactive peptides is the trypsin inhibitor isolated from tamarind seeds (TTI), which has shown potential effects in the context of obesity and its complications.^23^ Studies using an experimental obesity model have demonstrated that TTI reduces food intake, lowers plasma levels of tumor necrosis factor-alpha,^24^ and decreases plasma leptin levels in Wistar rats when compared to untreated rats.^25^ Furthermore, TTI has been shown to be nontoxic and provides a protective effect on the intestines, reducing villous atrophy—an injury characteristic of obesity models—highlighting its potential therapeutic use.^26^ Moreover, a recent study investigated in silico interactions between peptides derived from the theoretical model of the purified trypsin inhibitor from tamarind seeds (TTIp) and Human Pancreatic Lipase (HPL). The results indicated that the peptide (PEP2) interacts with the experimental structure of HPL near the active site, suggesting its potential as a novel HPL inhibitor.^27^

Given these findings and the growing interest in bioactive peptides as enzyme inhibitors, investigating the effect of isolated TTI on lipase activity has become a promising way for research. This study, which includes both in vitro and in vivo experiments, aims to contribute to the scientific and technical knowledge necessary for the development of bioactive proteins as adjuncts in the treatment of obesity and its complications. Considering the limited information about the functional properties of food proteins related to lipase inhibition, this research is crucial for a better understanding of the therapeutic potential of this molecule.

## 2 Methods

### 2.1 Obtaining the Trypsin Inhibitor Isolated from Tamarind Seeds (TTI)

The tamarind fruit was purchased from a local market in the city of Natal-RN, Brazil. The botanical identification was conducted by the Brazilian Institute of Environment and Renewable Natural Resources (IBAMA) in Natal/RN (Brazil) and registered in the National System for the Management of Genetic Heritage and Associated Traditional Knowledge (SisGen) under the number AF6CE9C.

The extraction of TTI followed the methodology described by Carvalho et al.^24^ The seeds were separated from the pulp and manually peeled using a stiletto knife to obtain the cotyledon. The peeled seeds were then ground using a domestic grain grinder (OSTER—OMDR 100) until a fine flour (40 mesh) was obtained. Next, the flour was mixed with 50 mM Tris–HCl buffer, pH 7.5, at a 1:10 (w/v) ratio under constant agitation (Magnetic Stirrer—Tecnal TE-081) for 3 h at room temperature. After stirring, the mixture was centrifuged (Centrifuge—BECKMAN COULTER, Avanti J-E) at 10,000 rpm for 30 min at 4 °C, yielding supernatant, referred to as the crude extract (CE).

Protein fractionation of the CE was performed via ammonium sulfate precipitation at saturation levels of 0–30% and 30–60%, resulting in fraction 1 (F1) and fraction 2 (F2), respectively. After each precipitation step, the samples were centrifuged under previously described conditions. The precipitates were resuspended in 50 mM Tris–HCl buffer, pH 7.5, and dialyzed in a 14 kDa pore membrane against distilled water for 3 days, with daily water replacement every 24 h. Subsequently, the samples were dialyzed against the same extraction buffer for two additional days, also with buffer replacement every 24 h.

The antitryptic activity of each fraction (F1 and F2) was assessed using *N*-benzoyl-l-arginine-*p*-nitroanilide (BApNa/1.25 mM)^28^ as a substrate. Activity was expressed as a percentage (%) relative to trypsin activity, and specific activity was determined. One inhibition unit (IU) was defined as the difference between the total enzymatic activity of trypsin and the activity of the enzyme in the presence of CE, protein fraction, and TTI, corresponding to an absorbance reduction of 0.01 at 410 nm. Thus, 1 IU was defined as the amount of inhibitor that reduced the absorbance by 0.01 optical density under the trypsin assay conditions. The specific trypsin inhibition activity was calculated by comparing the residual enzyme activity to the hydrolysis activity in the absence of the inhibitor (100% enzymatic activity).

The fraction with the highest antitryptic activity (F2) was applied to a Trypsin-Sepharose affinity column (CNBr-activated Sepharose 4B, GE Healthcare), pre-equilibrated with 50 mM Tris–HCl buffer, pH 7.5. Unbound proteins were eluted with the same buffer, while retained proteins were eluted with 5 mM HCl and collected in 5 mL aliquots. The protein profile was determined by spectrophotometry (UV/Visible spectrophotometer HACH DR 5000) at 280 nm. The fractions obtained from affinity chromatography were dialyzed against distilled water for 72 h, with water replacement every 24 h, then lyophilized and stored at −20 °C for further procedures.

The purity and molecular weight of the isolated protein were verified by discontinuous and denaturing polyacrylamide gel electrophoresis (SDS-PAGE) according to Laemmli.^29^ Molecular weight estimation of the isolated protein was performed using the Fisher BioReagents EZ-Run Rec Protein Ladder (Thermo Scientific), which contains proteins of 200, 150, 120, 100, 85, 70, 60, 50, 40, 30, 15, and 10 kDa. Protein quantification was carried out using the Bradford method,^30^ with bovine serum albumin (BSA) as the standard (Figure 1).

**Figure 1:**
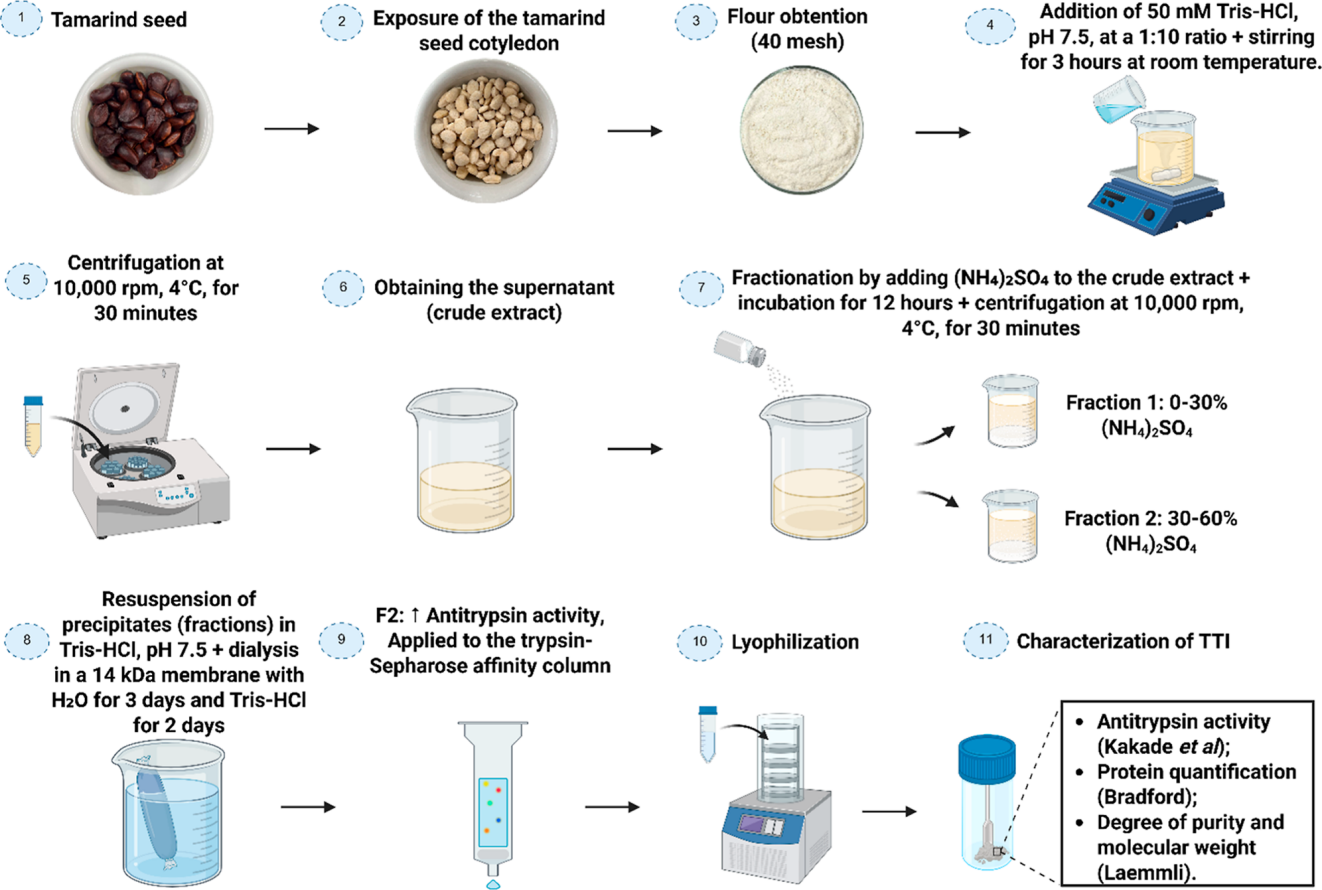
TTI isolation steps.

### 2.2 Inhibition Test against In Vitro Porcine Pancreatic Lipase

The inhibitory activity of pancreatic lipase was evaluated based on the procedures described by Jia et al.,^31^ with modifications, using 4-methylumbelliferyl oleate (4-MU) (Cayman Chemical, 18323-58-5) as a substrate. Initially, both Orlistat (Sigma, L3126, 9001-62-1), used as a positive control, and 4-MU were dissolved in dimethyl sulfoxide to prepare a stock solution. These solutions were subsequently diluted in PBS (pH 7.4) in a 9:1 ratio to obtain different concentrations.

For fluorescence assays, 25 μL aliquots of TTI at different concentrations (0.25, 0.125, 0.0625, 0.0312, and 0.0156 mg/mL) and 25 μL of porcine pancreatic lipase solution (0.075 mg/mL), both dissolved in PBS (pH 7.4), were added to 96-well microplates. The plates were incubated at 37 °C for 15 min. Then, 50 μL of 4-MU solution (0.0375 mM) was added to initiate the enzymatic reaction. The microplates were maintained at 37 °C for 20 min, after which 100 μL of 0.1 M sodium citrate (pH 4.2) was added to stop the reaction. The same procedure was performed with the standard inhibitor (Orlistat) at the same concentration used for TTI.

Each concentration of TTI and Orlistat was evaluated in triplicate. Absorbance values in the wells were measured using a microplate reader (BioTek, Synergy LX Multi-Mode Reader) with an excitation wavelength of 360 nm and an emission wavelength of 460 nm. The percentage of lipase inhibition was calculated according to the following equation:

1 $$inhibition\;\left(\%\right)=\left[\frac{\left(A_{c}-A_{s}\right)}{A_{c}}\right]\times 100$$

(*A*_c_, control absorbance; ss, sample absorbance)

### 2.3 Determination of IC_50_ and the Inhibition Constant (*K*_i_) against Lipase

Lipase activity was assessed in triplicate using increasing concentrations of TTI (0.0156, 0.0312, 0.0625, 0.125, and 0.25 mg). Subsequently, GraphPad Prism 8 software was used to generate a curve correlating the percentage of inhibition with the different concentrations, allowing the determination of the TTI concentration required to inhibit 50% of the enzyme activity. The correlation was established using nonlinear regression with log10-transformed values, and the response frequency was expressed as the percentage of inhibition.

The inhibition constant (*K*_i_) was determined using graphical analysis based on the Dixon and Webb model,^32^ where the *x*-axis corresponds to TTI concentrations and the *y*-axis represents the inverse of maximum reaction velocity. The *K*_i_ assay was carried out through a lipase inhibition assay using increasing concentrations of TTI and two concentrations of the specific lipase substrate. *K*_i_ was determined by measuring the release of 4-methylumbelliferone from 4-methylumbelliferyl oleate (4-MU).^33^ The *K*_i_ value was estimated from the intersection point of the two trend lines corresponding to the substrate concentrations.

### 2.4 In Vivo Experimental Model

#### 2.4.1 Animals and Husbandry

This study used 225 adult zebrafish (*Danio rerio*). Wild-type short fin, ∼5 months, sex ratio 1:1 male/female, 2.5 ± 0.2 g) obtained from a local supplier in Natal/RN and maintained in the FishLab vivarium (Physiology and Behavior Department, UFRN). The fish underwent a 10-day acclimation period to the laboratory conditions before the start of the feeding regime to induce weight gain. They were housed in 30 L tanks at a density of ∼3 fish/L, maintained in a closed circulation system in which temperature, pH, osmolarity, and dissolved oxygen were measured daily and corrected when needed (average values: 28 °C, pH 7.2, 600 μS, O_2_ 6 mg/L). Low levels of ammonia and nitrite were guaranteed by a daily water exchange. The photoperiod was set at 14:10 light/dark cycle (light on at 6 am, 250 l×), following the guidelines described by Westerfield.^34^

Experiments were approved by the Ethics Committee on the Use of Animals (CEUA) of the Federal University of Rio Grande do Norte under protocol number 022/2024 and certificate number 397.022/2024, following the guidelines of the National Council for Animal Experimentation Control (CONCEA, Brazil) and in accordance with the ARRIVE guidelines (Animal Research: Reporting of In Vivo Experiments).^35^ The use of animals complied with the 3Rs principle of sustainability: Replacement, Reduction, and Refinement.^36^

For obesity induction, fish were distributed in 15 12L-tanks (15 fish per tank) in a closed recirculating system rack. Obesity was induced by overfeeding with *Artemia* sp. at a rate of 60 to 120 mg per fish per day for 11 weeks. Twelve tanks received overfeeding. Eutrophic fish (3 tanks) received the same diet for the same duration but at a lower amount (15 to 75 mg per fish per day), ensuring proper growth and development. Feeding was administered in three portions per day with a 3 h interval between them, with weekly increases of 5.5 mg. All food was added directly to the fish tanks after closing the water income, ensuring that all animals could feed.

After completing obesity induction, all fish were classified according to the Body Condition Score (BCS) for adult zebrafish, allowing differentiation between eutrophic and obese animals (Figure 2).^37^ Obese fish were randomly assigned to four groups of 45 animals each, while eutrophic fish were assigned to a separate group (45 animals).

**Figure 2:**
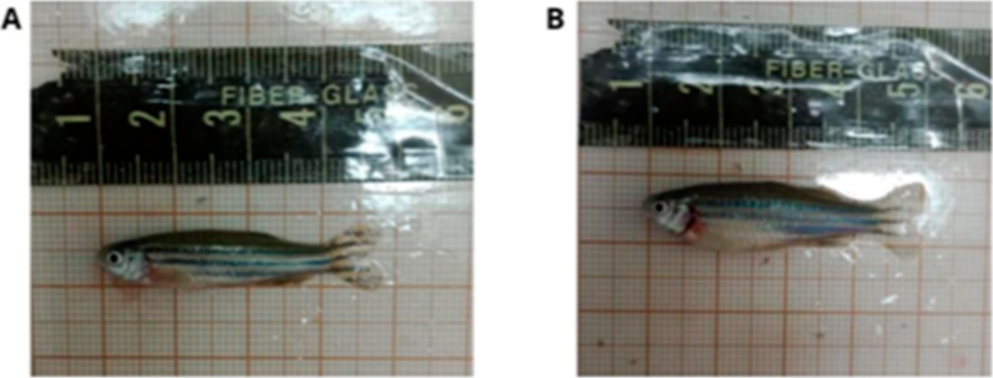
Classification of body condition in zebrafish based on the BCS method. (A) Zebrafish classified as eutrophic, with a body slightly wider than the head and a mildly convex ventral surface. (B) Zebrafish with obesity induced by overfeeding, exhibiting a body significantly wider than the head and a markedly convex ventral surface.

#### 2.4.2 TTI Treatment

By the end of the obesity induction period, the tanks containing obese and eutrophic fish were assigned to the following treatments (Figure 3).

1 Eutrophic and normally fed animals (EN) without treatment (*n* = 45): Received *Artemia* sp. (75 mg wet weight/fish/day).
2 Obese and hyperfed animals (OH) without treatment (*n* = 45): Received *Artemia* sp. (120 mg wet weight/fish/day).
3 Obese and hyperfed animals (OH) treated with Orlistat (*n* = 45): Received *Artemia* sp. (120 mg wet weight/fish/day) + 50 mg/kg of Orlistat. This group was considered the conventional treatment group.
4 Obese and hyperfed animals (OH) treated with TTI (*n* = 45): Received *Artemia* sp. (120 mg wet weight/fish/day) +25 mg/L of TTI.
5 Obese and normally fed animals (ON) treated with TTI (*n* = 45): Received *Artemia* sp. (75 mg wet weight/fish/day) +25 mg/L of TTI.

**Figure 3:**
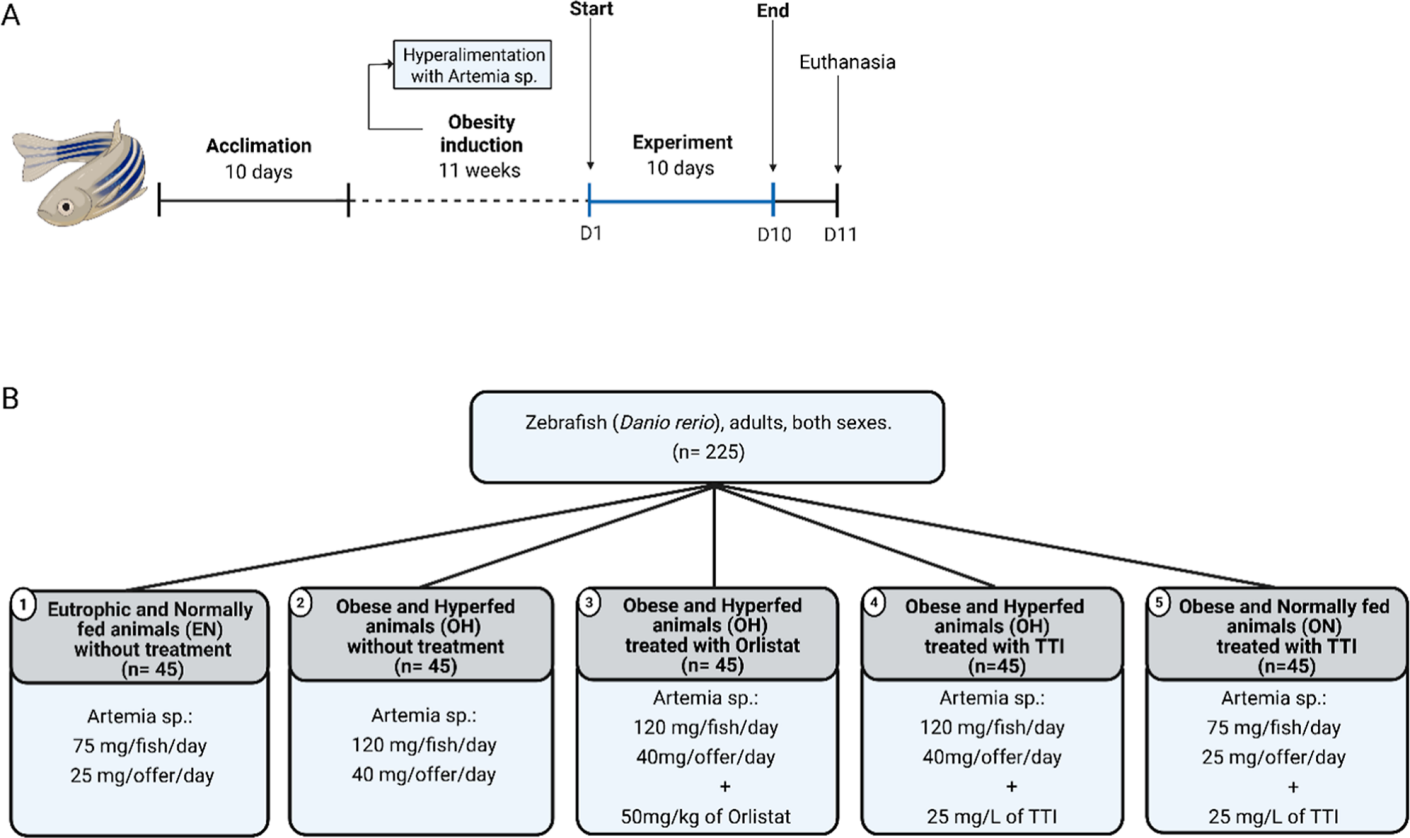
Experimental design. (A) Timeline depicting key events in the experimental protocol. (B) Division of experimental groups. EN: eutrophic and normally fed animals; OH: obese and hyperfed animals; ON: obese and normally fed animals; TTI: Trypsin inhibitor isolated from tamarind seeds.

The treatment phase lasted 10 days, a period established based on previous studies (22,79,80). The concentrations of TTI (25 mg/L) and Orlistat (50 mg/kg), the latter used as a positive control due to its role as a standard pancreatic lipase inhibitor, were determined based on the studies by Santos et al.^38^ and Ortega-Pérez et al.,^39^ respectively.

Every morning, in a fasting state, all animals, including those in the control groups, were transferred to 4 L beakers filled with a solution containing TTI, Orlistat, or nothing (control). TTI was diluted in 500 μL of filtered water, while Orlistat was initially dissolved in 50 μL of DMSO and then further diluted in 450 μL of filtered water and added to the beakers. The animals were exposed to the treatments for one h before being returned to their original tanks. The first feeding of *Artemia* sp. was administered 20 min after their return, followed by the second and third feedings at 3 h intervals.

#### 2.4.3 Lipase Activity

Fifteen fish from each group were euthanized by immersion in ice-cold water (0–4 °C) until complete immobilization, followed by immersion in clove oil (40 mL/L).^40^ After euthanasia, the fish were individually dissected, and the entire gastrointestinal tract was extracted and weighed. Pools of 5 intestines (3 pools per treatment) were homogenated and prepared according to the methodology of Mohammadi Arani et al.,^41^ with some modifications. The intestines were first homogenized with PBS (1:8, w/v) (pH 7.4) using an ultrasonic homogenizer (Fisherbrand, FB120) for 2.5 min, with 30 s intervals on ice. The homogenate was centrifuged (Centrifuge 5804R, Eppendorf) at 5,000 rpm for 25 min at 4 °C. The resulting supernatant was stored at −20 °C for the determination of lipase enzymatic activity.

The soluble protein concentration in the enzymatic extracts from all groups was quantified using the Bradford method^30^ with bovine serum albumin as the standard to normalize protein levels for lipase activity assays. Lipase activity was determined following the method described by Jia et al.,^31^ using 4-methylumbelliferyl oleate (4-MU) as the substrate. Enzymatic activity was assessed by measuring the release of 4-methylumbelliferone, the reaction product, following substrate hydrolysis by the homogenate. Absorbance values were recorded using a fluorescence microplate reader (BioTek Synergy LX Multi-Mode Reader) with an excitation wavelength of 360 nm and an emission wavelength of 460 nm.

To compare enzymatic activity across experimental groups, lipase activity was expressed as a percentage relative to the eutrophic group, which served as a control. The eutrophic group was set as the reference with 100% enzymatic activity. First, the mean absorbance of each experimental group was calculated, with blank values subtracted to obtain residual activity. The percentage of lipase activity for each group was determined using the following formula:

2 $$lipase\;activity\;\left(\%\right)=\left(\frac{residual\;activity\;of\;the\;experimental\;group}{residual\;activity\;of\;the\;eutrophic\;group}\right)\times 100$$

#### 2.4.4 Weight Assessment

Zebrafish weight measurements were taken on the first and last days of the experiment, before and after treatment administration, respectively. Initially, fish from all groups were transferred from their original tanks by using a 4 L beaker and individually placed into a smaller 50 mL beaker for weighing. Measurements were recorded using a precision digital scale (Ulela, Pro-50).^42^ After data collection, the mean and standard deviation of the weights for each group were calculated to analyze the weight variations throughout the treatment period.

#### 2.4.5 Lipid Profile

To analyze total cholesterol (TC), very low-density lipoprotein (VLDL), low-density lipoprotein (LDL), high-density lipoprotein (HDL), and triglycerides (TG), 15 whole-body fish per group were used, divided into 3 pools of 5 fish. Fish homogenates were prepared as described by Silva et al.,^42^ with some modifications.

Initially, the animals were euthanized under the conditions described in the previous section. The tail and head were removed, and the remaining bodies were cut into small pieces using scissors. These tissue fragments were placed in 15 mL Falcon tubes, and 2 mL of PBS buffer (pH 7.4) was added. The fish bodies were homogenized using an ultrasonic homogenizer for 1 min until the tissue was completely disintegrated. The rotor was rinsed with an additional 2 mL of PBS (pH 7.4), yielding a final volume of 4 mL of PBS (pH 7.4). The mixture was vortexed for 30 s and centrifuged at 5,000 rpm for 15 min at 22 °C. After centrifugation, the supernatant (plasma) was collected using a pipet and transferred to a microcentrifuge tube.

All biochemical analyses of plasma lipids were performed using commercial kits according to the manufacturers’ protocols: Labtest (Minas Gerais, Brazil) for TC, HDL, LDL, and VLDL, and Analisa (Belo Horizonte, Minas Gerais, Brazil) for TG.

### 2.5 Statistical Analysis

Data were expressed as the mean ± SD from three independent determinations. The results obtained from the inhibition tests were evaluated using a two-way ANOVA to determine the effects of the inhibitor concentration and inhibitor type on lipase inhibition. Multiple comparisons between groups were performed using Bonferroni’s multiple comparison test.

The sample size for the in vivo experiment was calculated based on a simple random sampling model (Cochran model),^43^ resulting in an “*n*” of 45 animals per group. The experimental data were tested for normality by using the Shapiro–Wilk test. Parametric variables were analyzed using a one-way ANOVA followed by Tukey’s post hoc test for multiple comparisons, whereas nonparametric variables were analyzed using the Kruskal–Wallis test followed by Dunn’s post hoc test to determine whether the lipid profile data differed among the tested groups. Furthermore, to assess the weight between the two time points (pre- and post-treatment), a paired *t*-test was performed.

Differences were considered statistically significant when the *p*-value was less than 0.05 (*p < 0.05; **p < 0.01; ***p < 0.001; ****p < 0.0001). Statistical analyses were performed using GraphPad Prism, version 5.0 (GraphPad Software, San Diego, CA).

## 3 Results

### 3.1 Isolation of the Trypsin Inhibitor from Tamarind Seeds

Protein fraction 2 (saturated with 30–60% ammonium sulfate) was subjected to affinity chromatography using Trypsin-Sepharose CNBr 4B to isolate TTI. The chromatographic column was calibrated with 50 mM Tris–HCl buffer, pH 7.5, and the adsorbed proteins were eluted with 5 mM HCl at a flow rate of 0.5 mL/min, yielding a trypsin inhibitory activity of 99.3% (Figure 4A). SDS-PAGE (12%) analysis revealed a predominant and enriched protein band with an approximate molecular mass of 20 kDa, confirming the isolation of TTI (Figure 4B).

**Figure 4:**
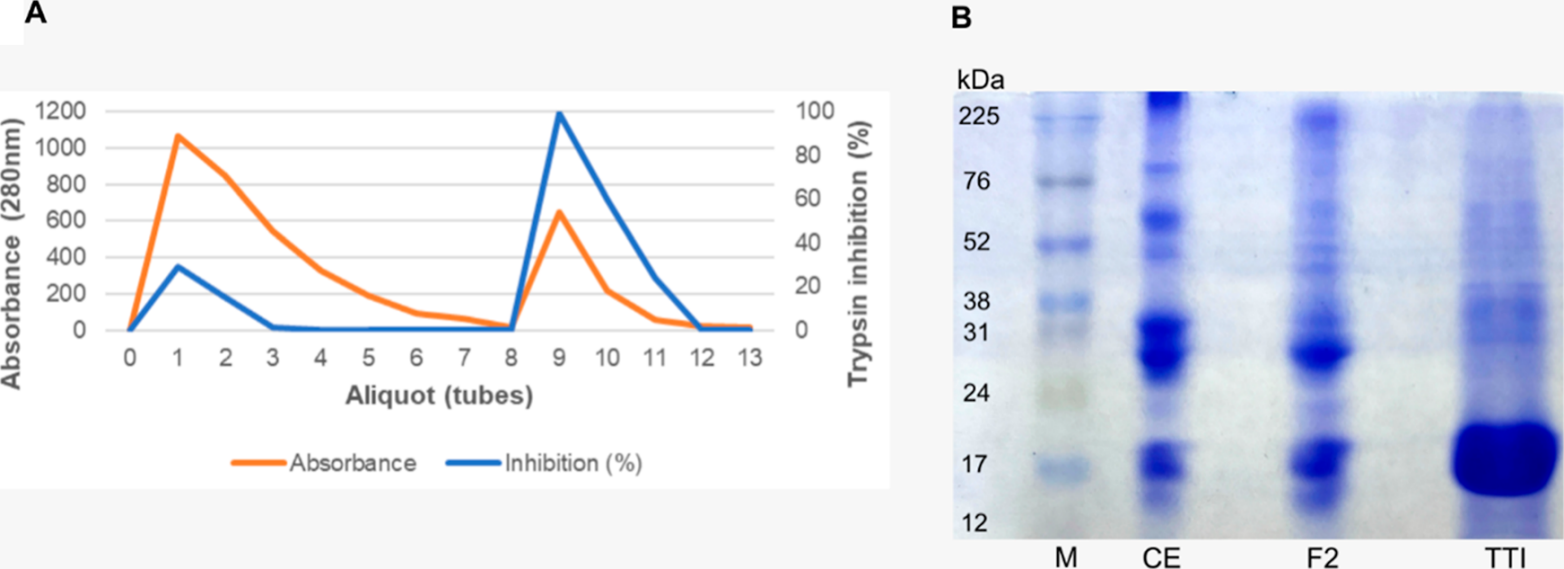
Isolation of trypsin inhibitor from tamarind seeds. (A) Chromatographic profile of protein fraction 2 obtained from the CE of tamarind seed flour (*Tamarindus indica L*) after affinity chromatography on Trypsin-Sepharose 4B CNBr. The column calibration was performed with Tris–HCl buffer (50 mM, pH 7.5), and the retained fraction was eluted from tube 9 onward using 5 mM HCl at a flow rate of 0.5 mL/min. Trypsin inhibitory activity was assessed using *N*-benzoyl-dl-arginine-*p*-nitroanilide (BapNa) as a substrate. (B) Discontinuous and denaturing polyacrylamide gel electrophoresis (SDS-PAGE) at 12%, stained with Coomassie blue. A prominent band is observed in the TTI lane near 20 kDa, consistent with the expected molecular weight of the isolated inhibitor, indicating successful isolation and enrichment of the protein of interest. M: Marker (Cytiva); CE: Crude extract; F2: Protein fraction saturated with 30–60% ammonium sulfate; TTI: Trypsin inhibitor isolated from tamarind seeds (10 μg/mL).

### 3.2 Inhibition Test against Porcine Pancreatic Lipase

In the in vitro lipase inhibition test, TTI showed lower inhibitory activity compared with the standard inhibitor Orlistat at all tested concentrations. At the lowest concentration evaluated (0.0156 mg/mL), TTI induced approximately 12% inhibition. As the concentration increased, the inhibition also rose, reaching around 37% and 83% at 0.0312 and 0.0625 mg/mL, respectively, the latter representing the highest level of inhibition observed. At higher concentrations (0.125 and 0.25 mg/mL), inhibition decreased slightly to 76% and 68%, respectively (Figure 5).

**Figure 5:**
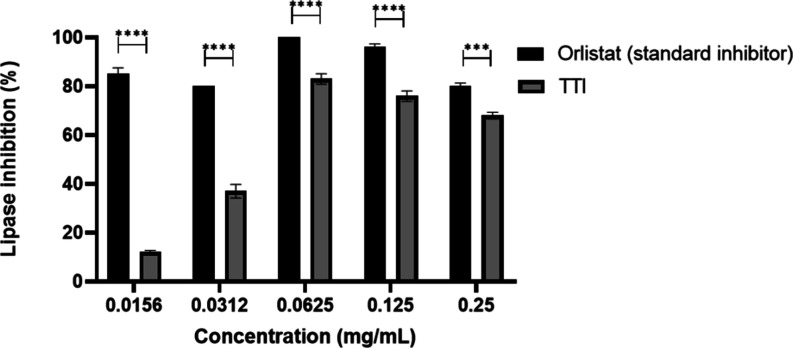
Comparison of lipase inhibition (%) by TTI and Orlistat at different concentrations. Inhibitory activity was assessed using 4-methylumbelliferyl (4-MU) as a substrate at a concentration of 0.0375 mM. A two-way ANOVA revealed a significant interaction between inhibitor concentration and inhibitor type (*F*–4, 11 = 205.7, DF = 4, *P* < 0.0001). The standard inhibitor showed significantly higher inhibition at all tested concentrations compared to TTI (Bonferroni post hoc, *P* = 0.0002 for 0.25 mg/mL, and *P* < 0.0001 for all other concentrations). TTI: Trypsin inhibitor isolated from tamarind seeds. Results represent the mean ± SD of two independent experiments with three replicates (***p < 0.001; ****p < 0.0001).

### 3.3 Determination of IC_50_ and Inhibition Constant (*K*_i_) against Lipase

The IC_50_ of TTI was determined using a lipase assay, yielding a value of 1.59 × 10^–9^ mol L^–1^ (30 μg/mL), indicating the concentration required to inhibit 50% of lipase activity (Figure 6A). Additionally, the *K*_i_ value was estimated as 2.38 × 10^–8^ mol L^–1^ (Figure 6B), classifying it as a noncompetitive inhibitor.

**Figure 6:**
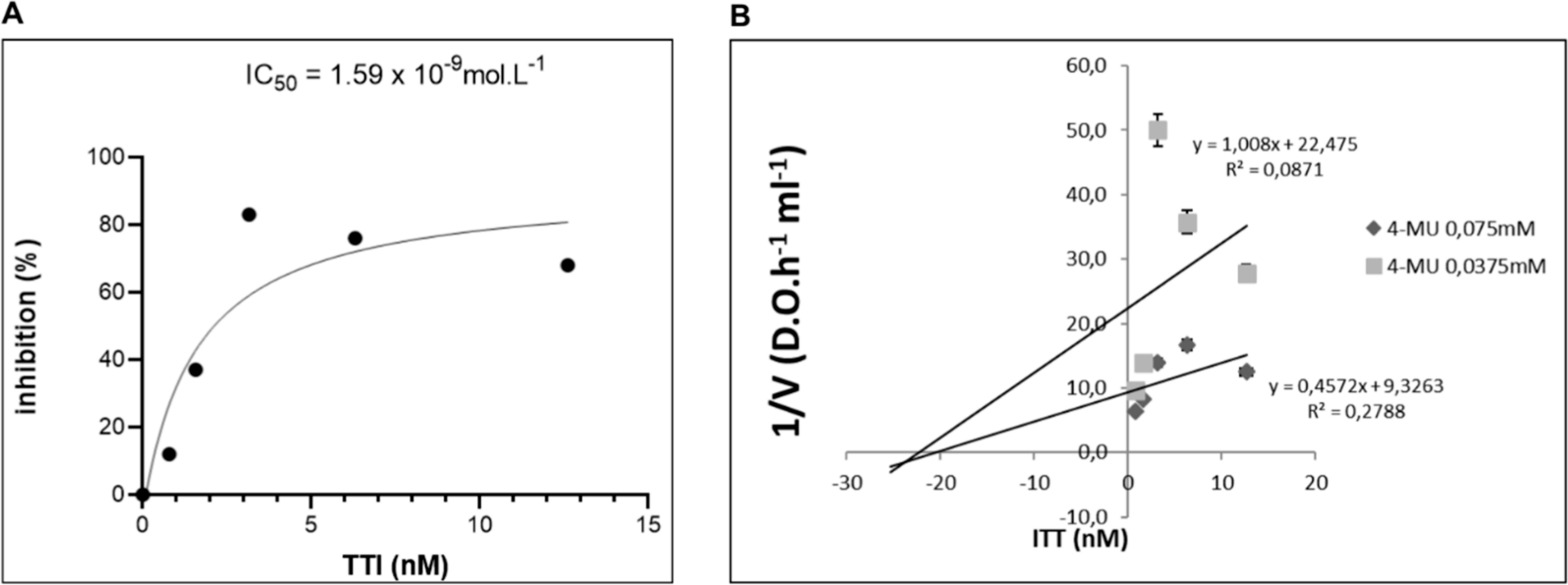
Determination of IC_50_ and *K*_i_ of TTI against lipase. (A) Lipase inhibition curve using increasing amounts of TTI (0.0156, 0.0312, 0.0625, 0.125, and 0.25 mg/mL). Inhibitory activity was assessed using 4-methylumbelliferyl (4-MU) as a substrate. (B) Estimation of inhibition kinetics and inhibition constant (*K*_i_) for lipase by TTI. IC_50_: amount of inhibitor that reduces lipase activity by 50%. TTI: Trypsin inhibitor isolated from tamarind seeds; 4-MU: 4-methylumbelliferyl.

### 3.4 Lipase Activity

The effects of different treatments on lipase enzymatic activity in zebrafish were evaluated, considering that the lipase activity in the eutrophic group was 100% (Figure 7). ANOVA analysis indicated a significant difference between the groups (*F* = 16.57, *p* = 0.0004). The Tukey post hoc test showed that zebrafish from the OH untreated, OH treated with Orlistat, and OH treated with TTI groups exhibited an increase in average lipase activity compared to EN–untreated.

**Figure 7:**
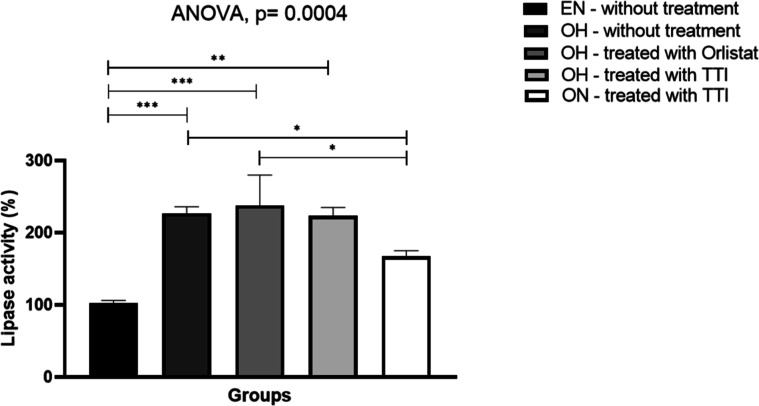
Lipase activity in zebrafish intestinal homogenate subjected to different treatment types for 10 days. Lipase enzymatic activity was expressed as a percentage for the experimental groups. Data normality was assessed using the Shapiro–Wilk test. Comparisons among groups were performed using one-way ANOVA, followed by Tukey’s post hoc test for multiple comparisons. EN: eutrophic and normally fed animals; OH: obese and hyperfed animals; ON: obese and normally fed animals; TTI: trypsin inhibitor isolated from tamarind seeds. Statistically significant values were considered for *p* ≤ 0.05 (*p < 0.05; **p < 0.01; ***p < 0.001).

Moreover, comparisons among the treated groups showed that, despite the increase in mean activity, there was no significant difference between OH treated with Orlistat and OH treated with TTI. A difference was observed only in zebrafish from the normally fed group (ON–treated with TTI), which displayed lower lipase activity. These findings indicate that treatments with Orlistat and TTI did not inhibit lipase activity in vivo under the tested conditions.

### 3.5 Weight Assessment

The variation in zebrafish weight over the 10 day treatment period was evaluated across different experimental groups. No significant weight difference was observed between the beginning and end of the experiment in the EN untreated (A), OH untreated (B), and OH treated with Orlistat (C) groups (*p* > 0.05). In contrast, zebrafish in the TTI-treated groups, both hyperfed (D) (*t* = 5.5, df = 2, *p* = 0.0314) and normally fed (E) (*t* = 6.84, df = 2, *p* = 0.0207), exhibited a significant weight reduction over the 10-day treatment period (Figure 8).

**Figure 8:**
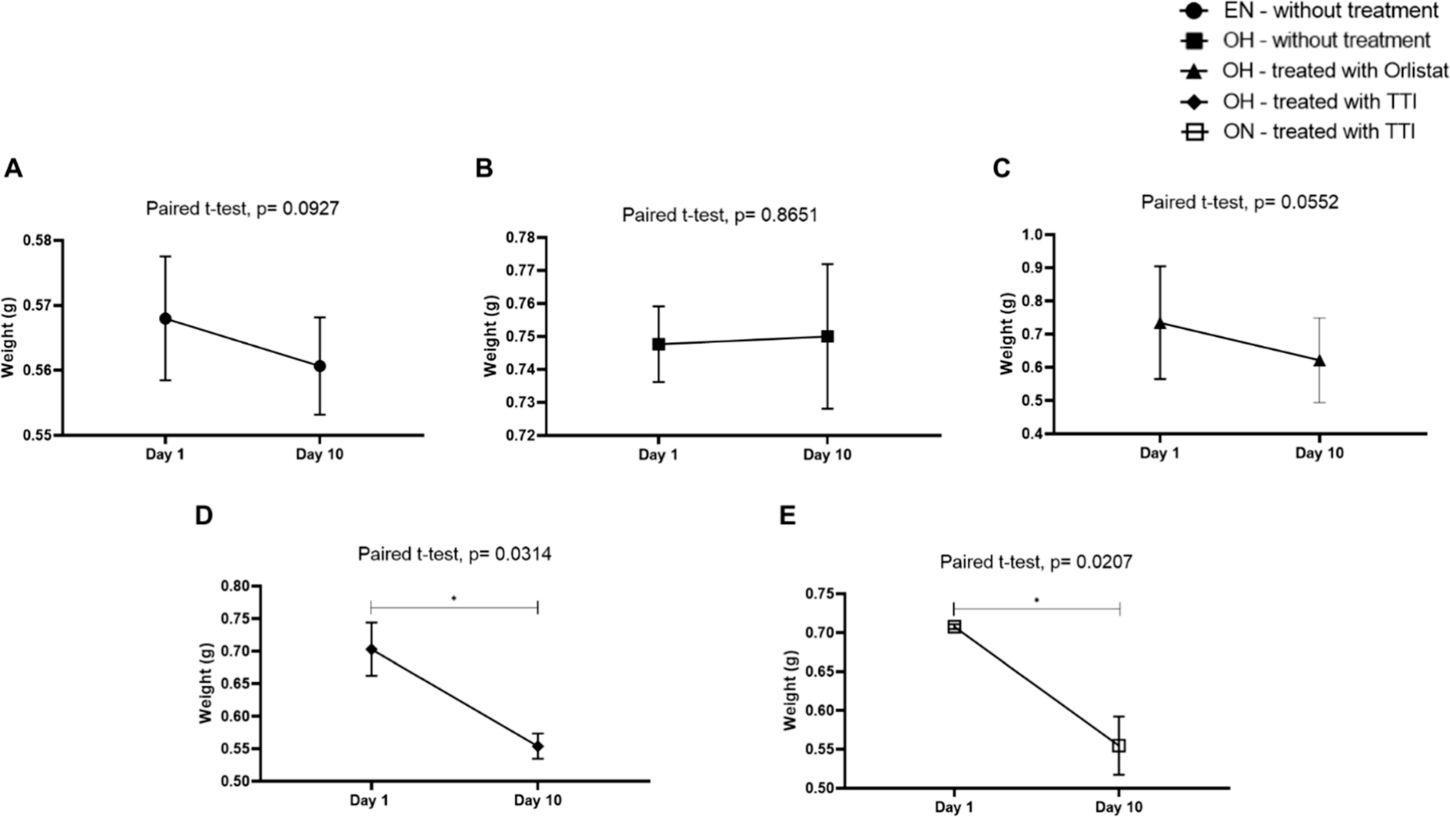
Variation in average weight of zebrafish between day 1 (start of the experiment) and day 10 (after treatment). (A) EN—without treatment; (B) OH—without treatment; (C) OH—treated with Orlistat; (D) OH—treated with TTI; and (E) ON—treated with TTI. The weight is expressed in grams (g), with values represented as the variation of the mean ± SD. A paired *t*-test was performed to compare weights between pre- and post-treatment conditions. EN: eutrophic and normally fed animals; OH: obese and hyperfed animals; ON: obese and normally fed animals; TTI: trypsin inhibitor isolated from tamarind seeds. Statistically significant values were considered for *p* ≤ 0.05 (*p < 0.05).

### 3.6 Lipid Profile

The lipid profile of zebrafish subjected to different treatments for 10 days revealed significant differences in plasma concentrations of VLDL, HDL, and triglycerides among the experimental groups. Plasma total cholesterol concentrations showed no significant difference between groups (Kruskal–Wallis test = 8.2, *p* = 0.0541), despite higher mean value in TTI-treated fish. VLDL concentrations were significantly higher in zebrafish from the hyperfed obesity groups (OH), both treated and untreated, compared to eutrophic and normally fed zebrafish (EN–untreated) (*F* = 16.19, *p* = 0.0002). LDL concentrations showed no significant differences (*F* = 2.979, *p* = 0.0736), remaining similar across all groups. However, HDL concentrations were significantly elevated in zebrafish from both TTI-treated groups (*F* = 17.74, *p* = 0.0003). Triglyceride concentrations were higher in zebrafish from the OH-untreated and OH-treated groups (*F* = 19.22, *p* = 0.0007) compared to zebrafish from the EN untreated group, which exhibited the lowest concentrations. However, triglyceride levels in zebrafish from the TTI-treated groups, regardless of feeding condition (normally fed or hyperfed), did not differ statistically (Figure 9).

**Figure 9:**
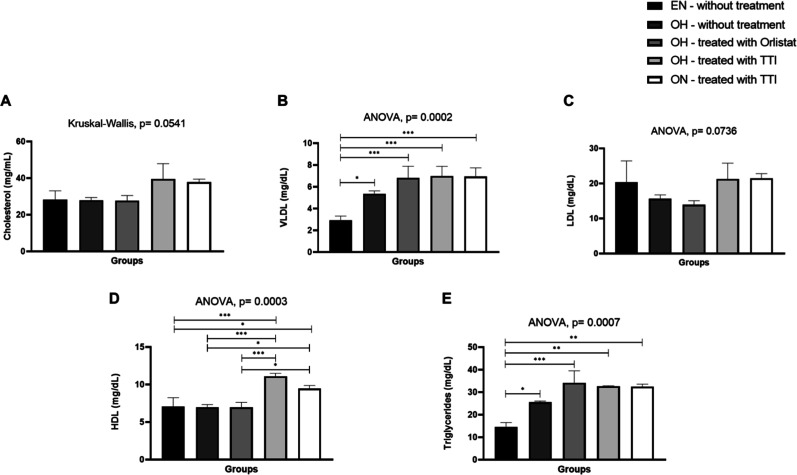
Lipid profile in zebrafish subjected to different treatment types for 10 days. (A) Total cholesterol; (B) VLDL; (C) LDL; (D) HDL; and (E) triglycerides. Values are expressed as mean ± SD. Data normality was assessed using the Shapiro–Wilk test. Comparisons between groups were performed using one-way ANOVA, followed by Tukey’s post hoc test for multiple comparisons. EN: eutrophic and normally fed animals; OH: obese and hyperfed animals; ON: obese and normally fed animals; TTI: trypsin inhibitor isolated from tamarind seeds; VLDL: very low-density lipoprotein; LDL: low-density lipoprotein; HDL: high-density lipoprotein. Statistically significant values were considered for *p* ≤ 0.05 (*p < 0.05; **p < 0.01; ***p < 0.001).

## 4 Discussion

The search for new antiobesity agents has intensified, as the availability and adverse effects of conventional medications limit their widespread use.^15,16^ Consequently, lipase inhibitors have emerged as strong candidates in this search, as they delay the absorption of dietary triglycerides, promoting a better energy balance.^20^

In this study, the trypsin inhibitor isolated from tamarind seed, termed TTI, exhibited a major protein band with an estimated molecular weight of approximately 20 kDa. Previous studies conducted by the NutriSBioativoS research group had already identified and described the isolation of TTI. Once again, in this study, isolation was performed as previously demonstrated by Ribeiro et al.,^44^ Carvalho et al.,^24^ and Costa et al.^25^ The results confirmed that the TTI obtained maintained the same antitryptic activity and molecular weight as reported in previous studies, validating the reproducibility of the isolation method.

The kinetic study provided insights into TTI’s enzymatic inhibition mechanism against lipase. The inhibition constant (*K*_i_), which reflects the inhibitor’s affinity for the enzyme, was determined to be 2.38 × 10^–8^ Mol using the Dixon and Webb plot.^32^ This finding suggests that TTI is a reversible noncompetitive inhibitor, as it binds to the enzyme and exerts its inhibitory effect regardless of whether the substrate is already bound.^45^ This type of inhibition was confirmed by the intersection of lines representing different substrate concentrations on the *x*-axis.^45^

The IC50 value obtained in this study (1.59 × 10^–9^ mol or 30 μg/mL) indicates that TTI has a strong inhibitory effect on lipase activity. For comparison, a study using protein hydrolysates from *Pisum sativum L*. reported an IC_50_ of less than 4,200 μg/mL.^19^ That finding highlights TTI’s promising potential beyond its previously reported trypsin inhibition, as it required significantly lower concentrations to achieve 50% inhibition of lipase activity. Notably, in this study, TTI was tested in its isolated form, and its efficiency under these conditions reinforces its potential for bioprospecting and future applications.

However, when comparing TTI’s lipase inhibition to that of Orlistat, the standard inhibitor demonstrated superior efficacy, likely due to its irreversible inhibition mechanism.^46^ Orlistat’s chemical structure includes a reactive β-lactone ring, which covalently binds to a serine residue in the catalytic triad of the pancreatic lipase active site.^47^ This covalent binding prevents lipase from hydrolyzing triglycerides, thereby blocking its enzymatic activity.^20^

In contrast, TTI exhibited dose-dependent inhibition, requiring higher concentrations to achieve efficacy similar to that of Orlistat. This can be attributed to TTI’s reversible inhibition mechanism, as mentioned earlier, which allows the enzyme to regain activity once the inhibitor is removed.^48^ A potential advantage of this mechanism is that TTI, by not acting irreversibly—i.e., not permanently inactivating the enzyme—may be associated with a lower incidence of gastrointestinal adverse effects, such as steatorrhea (reported in approximately 30% of patients treated with Orlistat), as well as symptoms like fecal urgency and oily leakage.^49^

However, when lipase activity was evaluated in intestinal homogenates of obese zebrafish, treatments with Orlistat and TTI did not inhibit the enzyme in vivo under the tested conditions. This result may be explained by the fact that, when administered in the tank water, both TTI and Orlistat were primarily absorbed systemically via gill permeation.^50^ In the case of Orlistat, its primary mechanism of action occurs in the lumen of the stomach and small intestine,^51^ with minimal systemic absorption,^52^ which may have compromised its efficacy in this experimental model. Therefore, when using zebrafish as a model, it is essential to include strategies such as oral administration via gavage to mimic physiological exposure.

Additionally, the animals were subjected to overfeeding during obesity induction, which may have contributed to increased pancreatic lipase expression.^53^ This upregulation of enzymatic activity could have necessitated higher drug doses to achieve effective inhibition. However, the administered amount may not have been sufficient, even considering Orlistat’s irreversible inhibition mechanism. The lack of inhibition observed in homogenates from zebrafish treated with TTI may be attributed to the dose-dependent nature of lipase inhibition. Thus, it is possible that the administered TTI concentration was insufficient to exert a significant effect on lipase activity in vivo.

The discrepancy between the in vitro and in vivo results may also be attributed to structural differences between the enzymes used in each assay. The in vitro test employed human pancreatic lipase, whereas the in vivo analysis involved the endogenous lipase of zebrafish, which may possess structural particularities that affect its affinity for and interaction with TTI.

On the other hand, the absence of lipase inhibition in the homogenates may explain why Orlistat-treated animals did not exhibit the expected weight loss. Orlistat-induced weight loss is known to be directly related to its ability to inhibit lipase.^1^ This result may have been influenced by the drug’s low absorption or administered dose, as previously discussed, or even by the short treatment duration. Preclinical studies have demonstrated significant weight reduction with Orlistat administration in treatment protocols lasting 6 to 13 weeks.^54–56^ Additionally, clinical studies have shown that weight loss with Orlistat becomes evident only after 2 weeks of therapy initiation.^52^ Conversely, in this study, TTI-treated animals showed significant weight reduction within just 10 days of treatment, highlighting the potential of this biomolecule even in the short term and independent of lipase inhibition.

It is important to emphasize that, since the study’s objective was to test TTI, with Orlistat serving only as a control, the experimental period was set at 10 days. This time frame was based on previous experiments demonstrating various bioactive properties of TTI within this treatment duration.^24,44,57^

As reported in the literature, trypsin inhibitors are proteins that may serve as alternatives for prolonging hormonal action, acting as secretagogues of cholecystokinin (CCK), a hormone involved in short-term satiety regulation.^58^ Research involving trypsin inhibitors extracted from tamarind and peanuts has indicated reduced food intake, leading to lower weight gain in norm nutritional animals.^44,59^

Thus, the weight reduction observed in TTI-treated animals in this study may also be associated with CCK secretion, as zebrafish regulate this hormone in response to dietary changes.^60^ Studies have also indicated that CCK plays multiple digestive roles in teleosts, including regulating pancreatic enzyme release, stimulating gallbladder contraction, controlling intestinal motility, and delaying gastric emptying, all of which contribute to reduced food intake.^61,62^ It is pertinent for future studies to do the analysis of gene expression related to anorexigenic signals, including CCK, POMC, leptin, and CART—to better understand the role of TTI in regulating appetite.

Interestingly, even animals subjected to overfeeding showed weight reduction. In this regard, considering the findings of Ribeiro et al.,^44^ combined with the results presented here, it can be inferred that TTI exerts weight-reducing effects on both eutrophic and obese animals—specifically in zebrafish—regardless of the caloric intake provided during treatment.

Regarding the lipid profile, treatments with TTI and Orlistat did not significantly affect plasma triglyceride and VLDL concentrations. Triglyceride and VLDL levels remained significantly higher in zebrafish from the OH groups, regardless of treatment, compared to those in the EN group without treatment. This result may be attributed to overfeeding with *Artemia* sp., which, despite being considered a nutritionally balanced food and recommended in guidelines for laboratory-reared zebrafish,^34,63^ has a high-fat content when provided in large quantities, promoting the development of dyslipidemia.^42^ High-fat diets increase the accumulation of free fatty acids in the liver, enhancing the synthesis and secretion of VLDL. These lipoproteins serve as the primary pathway for the liver to export excess triglycerides, which originate from both free fatty acids in plasma and chylomicron remnants.^64^ Previous studies on *Artemia* sp.-induced obesity in zebrafish have also reported increased triglyceride levels.^42,65^

Furthermore, no statistically significant differences were observed in total cholesterol and LDL concentrations among the TTI-treated groups, corroborating the findings of Carvalho et al.^66^ In that study, obesity was induced over 17 weeks using a high-glycemic index and high-glycemic load diet, followed by a 10 day experimental period during which Wistar rats were treated with 730 μg/kg of TTIp.^66^

On the other hand, the analysis of the effects of TTI on the lipid profile revealed a significant difference in plasma HDL concentrations among the zebrafish groups. The TTI-treated groups exhibited significantly higher HDL levels compared to all other groups regardless of feeding regimen (hyperfed or norm nourished). This finding represents a novel contribution, as in the study by Carvalho et al.,^24^ a discrete increase in mean HDL concentration was observed in obese Wistar rats treated with TTI, although without statistical significance. Subsequently, in another study where TTI was administered in a nano encapsulated formulation (based on chitosan and isolated whey protein), a significant increase in plasma HDL concentrations was observed in obese Wistar rats. In this case, the improvement was likely due to the gradual release of TTI in the gastrointestinal tract, a characteristic conferred by encapsulation.^67^

It is well-known that HDL is one of the most important molecules in cardiovascular disease prevention due to its multiple anti-inflammatory, antiatherogenic, and antioxidant properties.^68^ Thus, this result, combined with the other findings presented here and in previous studies on TTI, suggests the potential for new applications of this biomolecule in the treatment of obesity and associated comorbidities. However, further studies are needed to optimize administration and dosage in preclinical models, aiming to confirm and expand the therapeutic potential.

## 5 Conclusion

The results of this study confirm that TTI was properly extracted and isolated, ensuring its quality for the analyses performed. Based on in vitro tests, TTI emerges as a promising candidate for obesity treatment. The results revealed a significantly lower IC_50_ compared with other lipase inhibitors described in the literature, reinforcing its enzymatic efficacy not only against trypsin but also against lipase. Additionally, the kinetic study revealed that TTI acted as a reversible noncompetitive inhibitor, a characteristic that may differentiate it from Orlistat and potentially reduce the gastrointestinal adverse effects associated with this drug. The in vivo findings provided further relevant insights. Although lipase inhibition was not observed under the tested conditions, TTI was effective in reducing the body weight of treated animals, suggesting its action through other metabolic pathways. These results highlight TTI as a multifunctional biomolecule with potential benefits for obesity management. Furthermore, the observed increase in HDL concentrations in TTI-treated fish reinforces its potential as a therapeutic alternative for the prevention of dyslipidemia and cardiovascular diseases.
